# Long-term body composition changes after bariatric surgery and their association with fat- and bone-derived hormones

**DOI:** 10.1007/s12020-026-04564-0

**Published:** 2026-03-09

**Authors:** Malgorzata M. Brzozowska, Sabina Galiniak, Dana Bliuc, Artur Mazur, John A. Eisman, Jerry R. Greenfield, Jacqueline R . Center

**Affiliations:** 1 Garvan institute of Medical Research, Darlinghurst, NSW, Australia; 2 Department of Endocrinology, Sutherland Hospital, NSW, Australia; 3 University of New South Wales Sydney, Faculty of Medicine, Sydney, NSW, Australia; 4 University of Rzeszow, Faculty of Medicine, Rzeszow, Poland; 5 University of Notre Dame Australia, School of Medicine Sydney, Sydney, NSW, Australia; 6 Department of Endocrinology, St Vincent’s Hospital Clinical School, Darlinghurst, NSW, Australia

**Keywords:** Bariatric surgery, Adiponectin, Osteocalcin, Body composition changes

## Abstract

**Objective:**

Bariatric surgery effectively reduces obesity-related comorbidities, however fat mass loss is often accompanied by lean mass reduction. This 36-month prospective study (N=55) examined body composition changes following three bariatric procedures compared with a diet-controlled group (Diet).

**Methods:**

Random intercept mixed-effects models assessed 36-month trajectories of fat and lean mass, comparing surgical groups with Diet, adjusting for age and sex. Associations between body composition changes, measured by dual-energy X-ray absorptiometry and adiponectin (fat-derived) and undercarboxylated osteocalcin (ucOC; bone-derived) hormones were evaluated at 0, 1, 3, 6, 12, 24, and 36 months.

**Results:**

Roux-en-Y gastric bypass produced the greatest fat mass loss (46.6%), followed by sleeve gastrectomy (30.1%) and laparoscopic adjustable gastric banding (20.5%). Corresponding lean mass losses were 14.2%, 11.7%, and 5.2%. In Diet changes in fat mass (−2.1%) and lean mass (+3.6%) were non-significant. Over 36 months, all surgical groups lost significantly more lean mass than Diet, with the greatest losses after RYGB (15.1%) and LAGB (14.8%). Women experienced 30% greater lean mass loss than men (95% CI: -34%, -26%). Each additional year of age was associated with a 0.2% lean mass decline (95% CI: -0.5%, 0%). After controlling for treatment procedure, age and sex, each twofold increase in adiponectin and ucOC was associated with 5.0% (95% CI: -9.4%, -2.4%) and 3.7% (95% CI: -5.0%, -1.3%) reductions in lean mass, respectively.

**Conclusions:**

These findings underscore the lean mass loss risk after bariatric surgery, particularly in women and older adults, and highlight the importance of long-term monitoring of body composition, nutrition and bone health.

**Clinical trial registration:**

The study was registered as ANZCTR: 12613000188730.

**Supplementary Information:**

The online version contains supplementary material available at 10.1007/s12020-026-04564-0.

## Introduction

Bariatric surgery is a well-established intervention designed to achieve significant weight loss and improve body composition in individuals with obesity [1]. This procedure leads to substantial reductions in both visceral and subcutaneous fat, contributing to the resolution or improvement of obesity-related comorbidities such as type 2 diabetes and cardiovascular disease. However, fat mass loss is invariably accompanied by a reduction in lean body mass, including skeletal muscle [2], which is concerning, given its key role in metabolic regulation, physical strength and functional independence.

Lean mass loss can be considerable, accounting for up to 20–30% of total weight lost—particularly in the early postoperative period—and may vary widely across different weight loss interventions, including dietary, pharmacological (e.g., glucagon-like peptide-1-based therapies), and surgical approaches [3]. In a comparison of 26 dietary/behavioural and 29 bariatric surgery cohorts, lean mass comprised 19.2% to 23.6% of total weight [4]. Excessive fat-free mass (FFM) loss is undesirable, as non-adipose tissues are essential for maintaining resting metabolic rate, thermoregulation, skeletal health, balance and quality of life, particularly with advancing age [5].

Emerging, although limited evidence, suggests that the hormonal and metabolic changes induced by bariatric surgery may disproportionately affect lean body mass with few trials including dietary control group. In particular, the interplay between hormones derived from bone and adipose tissue may influence lean mass regulation during rapid weight loss. Adipocytes and osteoblasts originate from a shared mesenchymal progenitor [6] and the skeleton itself has been increasingly recognised as an endocrine organ with bone cells ‒ osteoblasts and osteoclasts ‒ secreting osteokines. These osteokines not only influence bone homeostasis but also play roles in energy and glucose metabolism [7, 8]. The osteocalcin is a non-collagenous bone matrix protein and marker of osteoblastic activity which plays key roles in bone mineralisation and calcium homeostasis [9]. In children and adolescents with obesity, serum osteocalcin is negatively correlated with BMI, body fat percentage, and waist circumference [10]. Osteocalcin also supports muscle physiology by promoting glucose and fatty acid uptake in muscle cells, potentially preventing sarcopenia [11].

In murine models, the undercarboxylated form of osteocalcin (ucOC) acts as a hormone with osteocalcin knock-out mice showing increased fat mass and decreased insulin sensitivity [7]. UcOC also promotes adiponectin production, contributing to reductions in fat mass and improvements in insulin sensitivity in both rodents and humans [7, 12].

Adiponectin, regulated by osteocalcin, improves insulin sensitivity, promotes fatty acid oxidation while inhibiting hepatic gluconeogenesis [13, 14]. Although adiponectin has been shown to support muscle regeneration via binding to T-cadherin, paradoxically, higher levels have been observed in individuals with sarcopenia [15, 16]. Previous research suggests that decreased sex hormone-binding globulin (SHBG) is inversely associated with overall and visceral adiposity and with other components of the metabolic syndrome [17]. Adiponectin increases plasma SHBG levels by upregulating hepatocyte nuclear factor-4α, which inhibits hepatic lipogenesis and promotes hepatic β-oxidation [18].

Given these complex hormonal interactions, we hypothesised that the type of surgical procedure significantly influences the extent of lean mass loss relative to the total body weight loss with more pronounced reductions expected following Roux-en-Y gastric bypass (RYGB) and sleeve gastrectomy (SG) compared to diet-based interventions. Furthermore, we hypothesized that lean mass loss is influenced by fat-derived hormones, such as adiponectin and SHBG, as well as osteoblast-derived hormones, including osteocalcin and ucOC with the more prominent effect after surgical procedures.

This study aimed to evaluate longitudinal changes in body composition (fat and lean body mass) after three types of bariatric surgery laparoscopic adjustable gastric banding (LAGB), SG and RYGB and to compare these outcomes with those in a diet-controlled group (Diet). Secondly, we investigated the association between bariatric surgery-induced changes in body composition and adiponectin, SHBG, osteocalcin, and ucOC. Additionally, we examined the effects of bariatric surgery on lipid profiles.

## Methods

We conducted a prospective, observational study on patients with obesity undergoing four weight-loss interventions: dietary program (study control group), LAGB, SG, and RYGB. Participants were recruited pre-intervention and followed for 3 years (surgical groups) or 2 years (Diet group). Given lack of long-term weight loss and hormonal changes in Diet, only surgical groups were assessed at 36 months with hormonal data being available for GS and RYGB groups. Approved by St Vincent’s Hospital Ethics Committee, the study was registered (ANZCTR: 12613000188730). This study is a secondary analysis of this prospective cohort, with partial protocol published, which examined 36-month trajectories of body composition and lipids and explored sex-, age-, and hormone-related determinants of lean and fat mass changes across RYGB, SG and LAGB procedures in comparison with dietary intervention. Inclusion criteria were age 18–70, Body Mass Index (BMI) > 35 kg/m² or > 30 kg/m² with obesity-related complications. Women were premenopausal or > 5 years postmenopausal. Exclusion criteria included pregnancy or the use of bone-active therapy [19]. Participants were allocated to their specific intervention groups according to their probability of diabetes remission as per DiaRem score criteria [20].

At all visits patients were asked to fill in a 3-day record of their food and supplements intake. The recorded data were analysed using Food Works Nutrition Software (Xyris), with an Australian food database to calculate energy, protein, fat, carbohydrate and calcium intake. We have estimated the % of protein intake for each individual study group over the study period.

The Diet group (controls) underwent a 2-year medically supervised Optifast^®^ VLCD™ program involving dietary and physical activity counselling. The intensive phase (1–2 months) provided < 800 kcal/day via three Optifast^®^ products, low-starch vegetables, and oil. In the maintenance phase, one Optifast^®^ product was combined with reintroduced low-calorie meals, totalling 1000–1200 kcal/day.

Surgical procedures followed standard techniques. Assessments occurred at 7 time points: baseline and 1-, 3-, 6-, 12-, 24-, and 36-months post-intervention. Anthropometric measures included weight (TANITA scale, Tokio, Japan, ± 0.1 kg) and height (stadiometer, ± 0.1 cm) and waist circumference. Body composition (lean mass, total fat) was measured by dual-energy X-ray absorptiometry (DXA, GE-LUNAR Prodigy, GE HealthCare Technologies, Inc., Chicago, Illinois, USA) at baseline, 6, 12, 24, and 36 months with previously published data on changes in BMD [19]. Although participants attended 1- and 3-month visits, DXA was not performed at these early time-points to reduce burden and maintain retention; routine body-composition imaging at our institution begins at 6 months.

All measurements were performed between 8 and 9 am and after a 12-hour overnight fast. Fasting biochemical measures included plasma adiponectin, osteocalcin, ucOC, SHBG, and lipids (total, low-density lipoprotein [LDL]), high-density lipoprotein [HDL]cholesterol, triglycerides). Lipid changes were analyzed in 36 subjects not on lipid-lowering agents or metformin. Some longitudinal changes in weight, adiponectin, osteocalcin, and SHBG were previously published [21, 22].

### Analytical assays

Total adiponectin was measured using an enzyme-linked immunosorbent assay (EZHADP-61 K, Millipore), with a sensitivity of 1.5 ng/mL and CVs < 2.4%. ucOC was measured by enzyme immunoassay (EIA kit MK118, TaKaRa) and osteocalcin by in-house radioimmunoassay (CVs 20% at 11 µg/L and 20% at 20 µg/L). SHBG was measured using an electrochemiluminescent immunoassay (Elecsys kit, Roche Diagnostics) with a CV < 4%. Serum lipids (total cholesterol, HDL, and triglycerides) were measured by enzymatic colorimetry (Roche; 490 nm and Wako; 550 nm) with inter- and intra-assay CVs < 10%.

### Statistical analysis

**Fig. 1:**
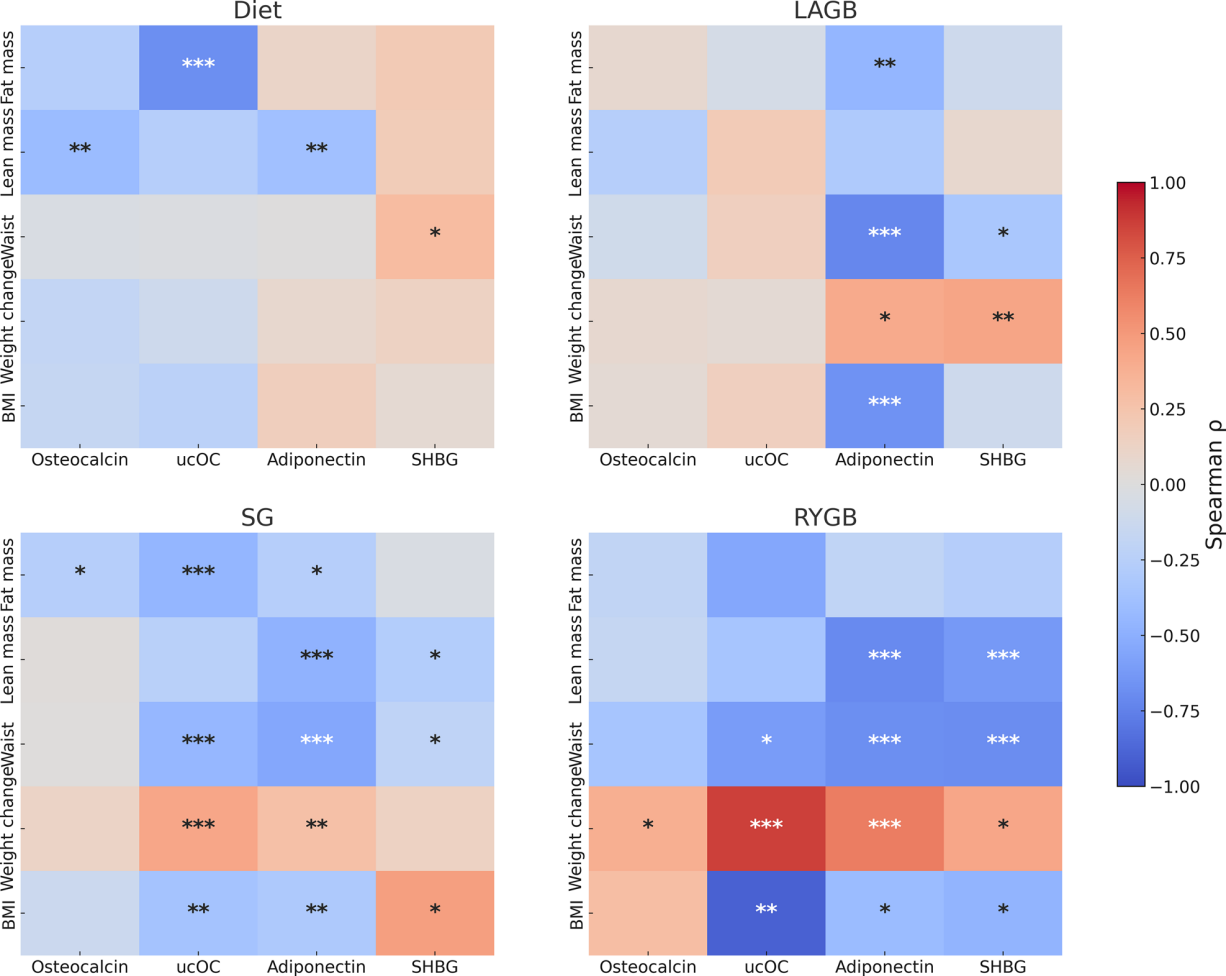
Spearman rank correlation heatmaps between hormonal markers and body composition indices across intervention groups. Rows represent body composition measures (fat mass, lean mass, waist circumference, weight loss, BMI), and columns represent hormonal markers (osteocalcin, ucOC, adiponectin, SHBG). The colour scale indicates the Spearman correlation coefficient (ρ), with warmer colours denoting positive and cooler colours denoting negative correlations. Asterisks indicate levels of statistical significance. Statistical significance: *p < 0.05; **p < 0.01; ***p < 0.001. Abbreviations: SG, sleeve gastrectomy; SHBG, sex hormone-binding globulin; LAGB, laparoscopic adjustable gastric banding; RYGB, Roux-en-Y gastric bypass; ucOC, undercarboxylated osteocalcin

The study enrolled 62 participants (15 per group target), with 7 withdrawals after baseline, (Supplementary Figure S1). It had > 90% power to detect an 8% difference in examined parameters between RYGB and Diet (α = 0.05)^(19)^. Normally distributed variables were summarised as mean (± SD) and non-normally distributed variables as median (IQR), with distribution assessed using the Shapiro–Wilk test. Baseline differences were evaluated using ANOVA with Tukey’s HSD post-hoc tests for normally distributed variables, and Kruskal–Wallis tests with Dunn’s post-hoc comparisons for non-normally distributed variables. Changes in weight and body composition were expressed as mean percentage change from baseline (95% CI). Associations between hormone concentrations and clinical parameters (weight, BMI, body composition) at each time point were assessed using Spearman’s rank correlation coefficients with Bonferroni-adjusted pairwise comparisons (Fig. 1). Missing dietary and hormonal data at 36 months in the Diet and LAGB groups were addressed using random intercept mixed-effects models which accommodate missing values in the outcome variable through maximum likelihood estimation.

Primary aim: Two random intercept mixed-effects models were used to assess 36-month trajectories of fat and lean mass, comparing all surgical groups with the Diet intervention, adjusting for age and sex. (covariates).

Secondary aim: Random intercept mixed-effects models examined individual hormone changes (adiponectin, osteocalcin, ucOC, SHBG) (exposure) as predictors of fat and lean mass loss (outcome variables) over 36 months.

All models were additionally adjusted for baseline fat/lean mass to account for baseline differences in initial body size and hormonal variables were log-transformed. A sensitivity analysis restricted to females with complete 24-month data was performed. Statistical significance was defined as *p* < 0.05. Analyses were conducted using STATISTICA (v13.3, StatSoft, 2017, Tulsa, Oklahoma, USA).

## Results

### Baseline characteristics

The study cohort consisted of 55 participants: RYGB (*n* = 7), SG (*n* = 21), LAGB (*n* = 11) and Diet-treated (*n* = 16). At baseline visit patients in the SG group had higher weights in comparison with Diet and LAGB groups and had greater waist circumferences and higher lean mass than LAGB. There were no baseline differences in levels of adiponectin, ucOC, osteocalcin and SHGB between groups (Table 1).

**Table 1:** Baseline characteristics

	Diet	LAGB	SG	RYGB	*P* value
Participants number	16	11	21	7	
Age (years)	54.5 (8.7)	**42.3 (13.1)** ^#^	51 (11)	51.1 (7.6)	**0.045**
Women, number (%)	10 (77)	10 (91)	12 (57)	5 (71)	0.23
Diabetes, number (%)	4 (31)	0	7 (33)	3 (43)	0.32
CVD, number (%)	1 (7)	1 (9)	1 (5)	0	0.84
Dyslipidemia, number (%)	6 (46)	6 (55)	11 (52)	4 (57)	0.76
Fat mass (kg)	47.53 (10)	51.73 (9)	56.11(8)	54.29 (13)	0.093
Lean mass (kg)	53.0 (11)	48.73 (8)	59.79 (9)*	53.57 (11)	**0.035**
Weight (kg)	109.4 (29)	110.91 (7)	**125.42 (17)** ^# *^	113.14 (16)	**0.0085**
BMI (kg/m^2^)	37.73 (7)	37.73 (5)	42.57 (5)	42.29 (8)	**0.024**
Waist (cm)	121.2 (15)	110.91 (7)	**132.33 (13** ^)*^	119.71 (12)	**0.0001**
Adiponectin (ng/mL): Median (IQ range)	6615 (4339–7609)	6407 (5152–9749)	6229 (4127–9339)	6029 (4318–11187)	0.97
Osteocalcin (µg/L)	8.95 (3.23)	8.03 (5.49)	8.26 (3.08)	7.64 (6.81)	0.27
ucOC (µg/L)	3.01 (2)	3.84 (2.15)	2.98 (2.06)	3.11 (0.94)	0.63
SHGB (nmol/L)	33.15 (14.71)	32.73 (13.41)	55.61 ± 57.52	22.15 (8.4)	0.49
Total cholesterol (mmol/L): Median (IQ range)	5.5 (4.5‒6.25)	4.5 (4.0‒5.3)	4.5 (4.2‒5.3)	4.8 (4.6‒4.8)	0.48
LDL cholesterol (mmol/L): Median (IQ range)	2.9 (2.1‒3.7)	3.2 (2.2‒3.9)	2.9 (2.1‒3.6)	3.05 (3‒3.1)	0.83
Triglycerides (mmol/L): Median (IQ range)	1.6 (0.9‒2.0)	1.0 (0.75‒1.6)	1.6 (0.95‒1.95)	1.3 (0.7‒5.1)	0.25
HDL cholesterol (mmol/L): Median (IQ range)	1.3 (1.25‒1.45)	1.2 (0.9‒1.3)	1.2 (1.0‒1.3)	1.2 (0.7‒1.4)	0.39

CVD, cardiovascular disease; SG, sleeve gastrectomy; SHBG, sex hormone-binding globulin; LAGB, laparoscopic adjustable gastric banding; RYGB, Roux-en-Y gastric bypass; ucOC, undercarboxylated osteocalcin

Data presented as mean (SD) unless stated otherwise, * statistically significant difference from LAGB group and # from diet groups (p < 0.05)

### Changes in weight over time

During the first 12 months, weight decreased by 4.3% (95% CI: -7.2%, 1.5%) in Diet, 14.2% (95% CI: -20%, -8.4%) in LAGB, 25.3% (95% CI: -29.2%, -21.5%) in SG, and 34.6% (95% CI: -42.7%, -26.6%) in RYGB. No significant changes occurred between 12 and 36 months (Table 2).

**Table 2:** Metabolic data at each time point

Variables	Baseline	1 month	3 months	6 months	12 months	24 months	36 months
Diet							
Weight (kg)	111.3 (28.25)	**110.2 (27.71)**	**105.4 (20.56)**	**104.7 (20.00**)	105.3 (23.82)	116.4 (38.51)	
BMI (kg/m^2^)	37.85 (6.79)	36.83 (5.41)	**35.69 (4.35)**	**35.23 (4.11)**	35.46 (4.72)	38.55 (7.78)	
Fat mass (kg)	48.37 (9.05)			42.75 (10.17)	44.42 (9.00)	47.35 (10.84)	
Lean mass (kg)	54.20 (10.15)			56.52 (11.19)	53.38 (9.87)	56.13 (10.13)	
Osteocalcin (µg/L)	8.72 (3.49)			5.71 (3.45)	6.64 (2.79)	7.47 (3.74)	
ucOC (µg/L)	3.0 (2.0)	3.15 (2.02)	2.18 (1.12)	2.87 (1.98)	2.65 (2.49)	4.06 (4.41)	
Adiponectin (ng/mL)	6960 (5028)	7549 (5955)	7307 (5121)	7906 (6672)	6947 (4405)	6186 (2617)	
SHBG (nmol/L)	33.15 (14.71)		47.38 (17.85)	41.86 (20.44)	39.13 (22.69)	39.32 (17.92)	
**LAGB**							
Weight (kg)	105.1 (16.34)	**98.15 (14.82)**	**93.97 (14.50)**	**91.89 (11.60)**	**88.82 (9.72)**	**90.00 (11.78)**	**85.39 (12.12)**
BMI (kg/m^2^)	37.73 (4.52)	**35.00 (4.38)**	**33.91 (4.09)**	**33.00 (3.55)**	**32.40 (2.91)**	**33.67 (3.64)**	**31.54 (3.39)**
Fat mass (kg)	51.22 (9.07)			**40.40 (8.21)**	**39.28 (6.33)**	**40.61 (7.72)**	**39.10 (8.24)**
Lean mass (kg)	50.52 (7.10)			**49.00 (6.49)**	**47.69 (6.80)**	**47.19 (7.41)**	**45.30 (7.60)**
Osteocalcin (µg/L)	8.36 (5.63)			9.45 (4.01)	10.29 (5.45)	9.55 (3.81)	
ucOC (µg/L)	3.84 (2.15)	3.65 (1.38)	4.59 (2.13)	4.13 (2.02)	5.59 (2.9)	4.12 (2.43)	
Adiponectin (ng/mL)	6863 (2346)	**8452 (2602)**	7775 (2898)	**8205 (2820)**	9022 (3349)	**11,491 (5973**)	
**SG**							
Weight (kg)	125.4 (16.79)	**111.0 (15.43)**	**103.6 (14.90)**	**95.63 (13.59)**	**93.00 (13.85)**	**92.69 (12.66)**	**93.62 (13.50)**
BMI (kg/m^2^)	42.57 (5.29)	**37.79 (4.95)**	**34.95 (4.35)**	**32.50 (3.97)**	**31.67 (4.20)**	**36.47 (23.07)**	**32.18 (3.38)**
Fat mass (kg)	56.54 (8.23)			**38.08 (9.34)**	**36.76 (11.26)**	**36.87 (9.06)**	**38.21 (7.69)**
Lean mass (kg)	60.23 (7.95)			**55.59 (7.52)**	**54.35 (7.77)**	**53.17 (8.10)**	**53.06 (8.58)**
Osteocalcin (µg/L)	8.27 (3.16)			**10.32 (3.97)**	**14.70 (6.41)**	10.86 (4.21)	**11.19 (3.21)**
ucOC (µg/L)	2.98 (2.06)	4.46 (2.11)	6.91 (7.75)	5.23 (1.56)	6.44 (4.63)	5.5 (4.38)	2.73 (1.55)
Adiponectin (ng/mL)	8839 (8522)	7929 (4248)	**8925 (3546)**	**10,272 (3909)**	**11,584 (5609)**	**14,301 (7733)**	**15,586 (8058)**
SHBG (nmol/L)	55.61 (57.52)		48.48 (10.31)	**92.62 (86.9)**	**77.28 (66.34)**	**79.1 (87.03)**	53.33 (31.24)
**RYGB**							
Weight (kg)	113.0 (15.91)	**97.65 (16.01)**	**87.56 (14.35)**	**79.20 (12.74)**	**73.69 (12.36)**	**74.57 (13.11)**	**74.99 (13.70)**
BMI (kg/m^2^)	42.29 (7.67)	**36.43 (6.95)**	**32.86 (6.64)**	**29.43 (5.59)**	**27.57 (4.47)**	**27.86 (4.81)**	**28.57 (4.24)**
Fat mass (kg)	54.88 (13.39)			**29.14 (11.23)**	**24.15 (7.81)**	**25.96 (8.42)**	29.10 (7.42)
Lean mass (kg)	54.28 (9.74)			**48.55 (9.80)**	**48.31 (10.23)**	**44.81 (9.18)**	**46.10 (10.04)**
Osteocalcin (µg/L)	5.47 (3.22)			**18.31 (7.72)**	**15.60 (5.50)**	**15.27 (7.22)**	
ucOC (µg/L)	3.11 (0.94)		**14.17 (5.65)**	10.58 (5.05)	10.54 (3.65)	7.54 (1.32)	
Adiponectin (ng/mL)	7835 (4203)	**9226 (3785)**	**9908 (3017)**	**10,647 (4226)**	**14,752 (6697)**	**20,096 (11017)**	19,318 (11884)
SHBG (nmol/L)	22.15 (8.4)			**53 (31.77)**	**54.33 (23.08)**	**53.5 (31.65)**	

Results presented as number, mean (SD) unless otherwise indicated. Include only patients who had at least 1 BMD data after entry. Bold type denotes statistically significant changes relative to baseline

SG, sleeve gastrectomy; SHBG, sex hormone-binding globulin levels; LAGB, laparoscopic gastric banding; RYGB, Roux-en-Y gastric bypass; UcOC, undercarboxylated osteocalcin

### Changes in waist circumference

All weight loss procedures led to significant reductions in waist circumference during the first 12 months: 5.3 cm (95% CI: -7.7, -2.8) in the Diet, 10.5 cm (95% CI: -16.7, -4.3) in LAGB, 21.2 cm (95% CI: -24.3, -18.0) in SG, and 23.8 cm (95% CI: -30.9, -16.7) in the RYGB group without further significant change after the first 12 months post interventions. Over 36 months, compared to Diet, on average patients who underwent LAGB had waist circumference reduction by 11.3% (95% CI: -16.3%, -6.4%), SG by 5.5% (95% CI: -9.3%, -1.5%) while RYGB resulted in reduction of 14.0% (95% CI: -19.5%, -8.5%).

### Changes in body composition

There were significant fat mass reductions in the first 12 months in all groups, followed by fat mass gain after the postoperative year as has been published previously (Table 2).

**Fig. 2:**
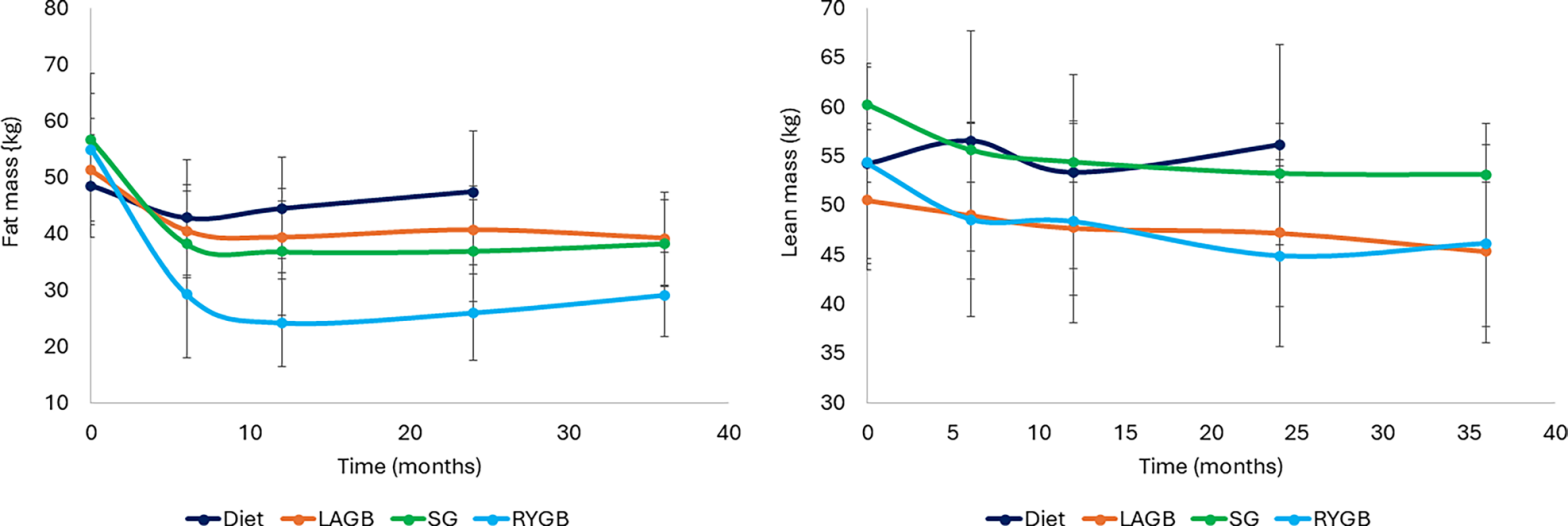
Body composition changes following weight loss procedures. The graph presents the mean and SD values over time. Abbreviations: LAGB, laparoscopic gastric banding; SG, sleeve gastrectomy, RYGB, Roux-en-Y gastric bypass

At 36 months, LAGB group lost 20.5% (95% CI: -32%, -3.6%) of fat mass, followed by SG group with 30.1% (95% CI: -36.8%, -23.4%), and RYGB group with 46.6% (95% CI: -56.5%, -36.6%), Fig. 2. RYGB patients achieved on average lower fat mass by 32% (95% CI: -45%, -19%) than Diet and this difference remained significant after controlling for participants’ age and sex.

Lean mass also decreased across all groups during the first 12 months: by 1.6% (95% CI: -3.4%, 0.2%) in the Diet, by 4.2% (95% CI: − 8.9%, − 0.6%) in LAGB, by 10.5% (95% CI: -13.3%, -7.6%) in SG, and by 10.3% (95% CI: -16.2%, − 4.4%) in the RYGB group (Fig. 2).

Between 12 and 24 months, all surgical groups experienced further lean mass loss, with a significant decrease of 4.3% (95% CI: -7.3%, -1.4%) in the RYGB group only. At 36 months LAGB lost ~ 5.0 kg at 5.2% (95% CI: -12.2%, 1.8%) of lean mass, followed by SG with ~ 7.0 kg at 11.7% (95% CI: -14.4%, -9.0%), and the RYGB group with ~ 7.6 kg at 14.2% (95% CI: -20.2%, − 8.2%) (Fig. 2).

Over 36 months, after adjusting for age and sex, all surgical groups lost significantly more lean mass compared to Diet: LAGB lost 14.8% (95% CI: -20%, -9.3%), SG 8.4% (95% CI: -13%, -3.7%), and RYGB 15.1% (95% CI: -20.2%, -9.3%). Sensitivity analysis: LAGB lost 12.6% (95% CI: -25.9%, -0.1%), SG 5.5% (95% CI: -16.8%, 5.7%), and RYGB 32.1% (95% CI:-45.6%, -18.2%). Standardised effect sizes for the adjusted 36-month differences between each surgical procedure and the Diet group for their lean mass changes yielded Hedges’ g values of 5.19 (95% CI 3.31, 7.07) for LAGB, 3.39 for SG (95% CI 1.51, 5.27) and 5.20 (95% CI 3.32, 7.08) for RYGB reflecting substantial physiological differences between surgical and dietary weight-loss methods.

The estimated proportion of lean mass loss relative to total weight loss increased over time, despite no further weight loss beyond the first 12 months. For SG, the proportion was 20.2% (95% CI: -27.6%, -12.8%) at 6 months, 22.6% (95% CI: -31.3%, -13.8%) at 12 months, 27.2% (95% CI: -36.4%, -18.1%) at 24 months, and 31.2% (95% CI: -42.6%, -19.8%) thereafter. Similarly, for RYGB, the proportion was 14.6% (95% CI: -21.6%, -7.6%) at 12 months, 17.8% (95% CI: -26.1%, -9.5%) at 24 months, and 21.5% (95% CI: -32.5%, -10.4%) subsequently. No statistically significant changes were observed in the LAGB or Diet groups.

After adjusting for the procedure and age, women lost significantly more lean mass with 30% greater reduction (95% CI: -34%, -26%) than men. Additionally, after adjusting for the procedure, each one-year increase in age was associated with an average lean mass loss of 0.2% (95% CI: -0.5%, 0%; *p* = 0.051).

The sensitivity analysis restricted to women with complete 24-month data (LAGB − 12.6%, SG − 5.5%, RYGB − 32.1%) demonstrated lean mass losses that were directionally consistent with the primary analysis. Notably, the magnitude of lean mass decline following RYGB (− 32%) was even greater than in the fully adjusted model, indicating that substantial postoperative lean mass loss persists when analyses are limited to women alone. Although SG group did not reach significance due to smaller sample size, the direction and magnitude were consistent with the primary analysis, reinforcing the robustness of these sex-related differences.

### Changes in lipid profile

At baseline, among 36 individuals not taking metformin or lipid-lowering medications, there were no significant differences between the four study groups in total cholesterol (*p* = 0.48), LDL cholesterol (*p* = 0.83), triglycerides (*p* = 0.25), or HDL cholesterol (*p* = 0.39) (Supplementary Table 1).

During the first 12 months, when compared to baseline, the Diet intervention showed no significant changes in lipid levels, whereas the surgical interventions produced beneficial effects. LAGB resulted in a modest reduction in total cholesterol (*p* = 0.028) and an increase in HDL cholesterol (*p* = 0.026). The SG procedure led to a decrease in triglycerides (*p* = 0.008) and an increase in HDL cholesterol (*p* = 0.001) within the first postoperative year, effects not significant in the RYGB group, likely due to its smaller sample size (Supplementary Table 1).

Over 36 months, SG patients maintained significant improvements in lipid profiles compared to baseline, with a reduction in median LDL cholesterol to 2.35 mmol/L (*p* = 0.04) and triglycerides to 0.85 mmol/L (*p* = 0.007), alongside an increase in HDL cholesterol to 1.5 mmol/L (*p* = 0.005). A similar pattern of improvement over time was observed in the RYGB group.

### Changes in protein intake over time

There was a small, non-significant increase in the proportion of protein (percentage) contributing to total caloric intake over time across all groups, reaching 22%±7% for Diet, 25%± 7% for LAGB at 24 months, and 21%± 4% for SG and 23%±5% for RYGB at 36 months.

### Hormonal changes over time

#### Undercarboxylated osteocalcin

**Fig. 3:**
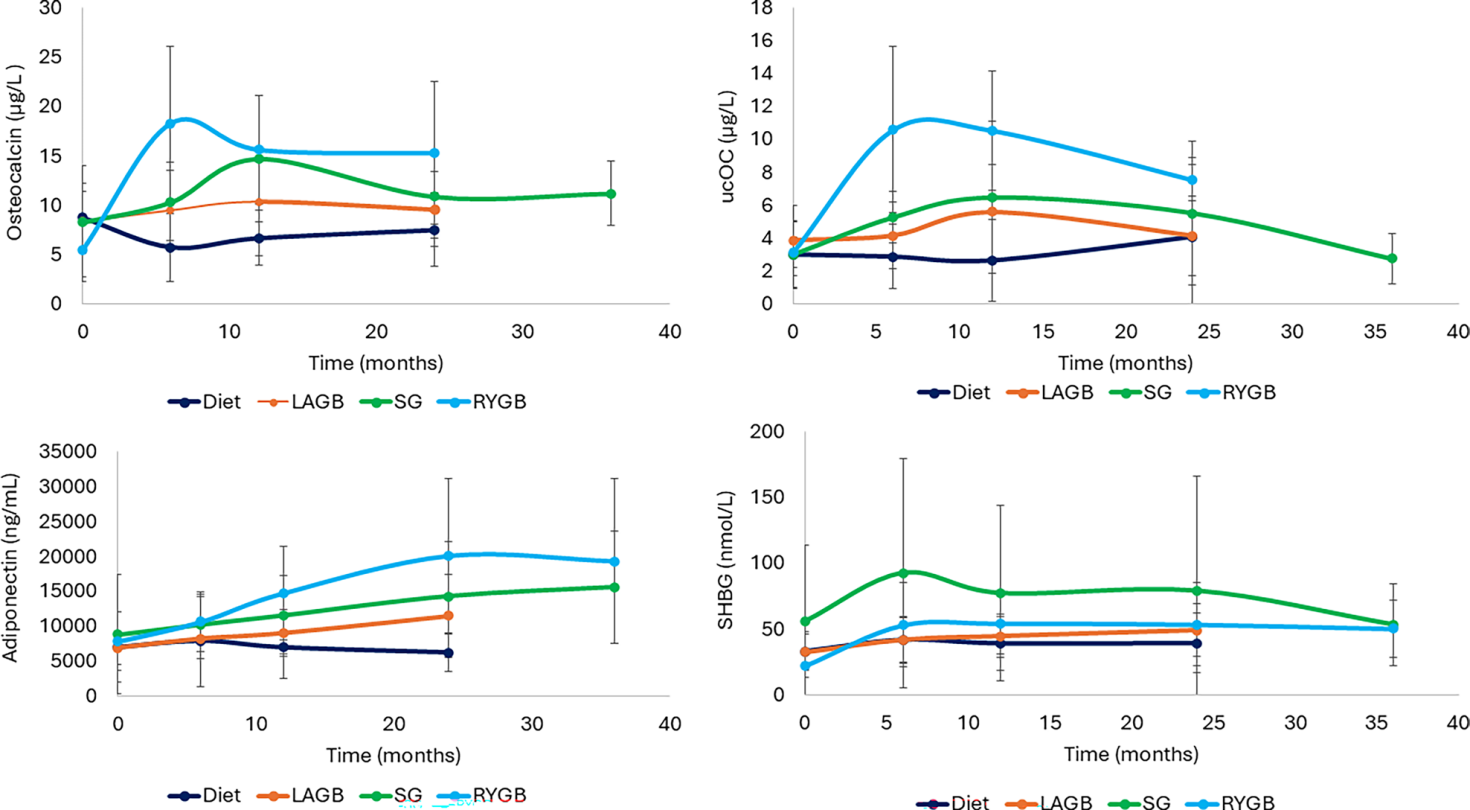
Changes in hormones following weight loss procedures. Abbreviations; LAGB, laparoscopic gastric banding; RYGB, Roux-en-Y gastric bypass, SG, sleeve gastrectomy. SHBG, sex hormone-binding globulin levels; ucOC, undercarboxylated osteocalcin

The pattern of ucOC change over time did not reach statistical significance in the surgical groups. However, an initial increase was observed in the first 12 months, followed by a decline of 37.3% (95% CI: -64.8%. -9.7%) in LAGB, 45.6% (95% CI: -72.0, -19.1) in SG, and 18% in the RYGB group (Fig. 3; Table 2). Compared with Diet, ucOC levels remained consistently higher at all time points, with an overall increase of 66% (95% CI: 18%, 113%) in LAGB and 53% (95% CI: 14%, 93%) in SG over 24 months. The RYGB group exhibited a more pronounced increase of 221% (95% CI: 164%, 277%) over 36 months.

#### Serum osteocalcin, adiponectin and SHBG

Changes in serum osteocalcin, adiponectin, and SHBG have been partially reported previously, showing increases over 36 months following SG and RYGB compared to both baseline and the Diet group. (Fig. 3; Table 2) [21, 22].

#### Relationships between changes in absolute values of weight, body composition and hormonal parameters

##### Adiponectin

Adiponectin correlated with weight loss in the LAGB (rho = 0.41, *p* = 0.011), SG (rho = 0.28, *p* = 0.0059), and RYGB groups (rho = 0.63, *p* = 0.00030) with an inverse association with fat mass in LAGB (rho = -0.46, *p* = 0.0078) and SG (rho = -0.26, *p* = 0.013) groups as well as with waist circumference in all surgical groups (Fig. 1).

Adiponectin also demonstrated an inverse association with lean mass in Diet (rho = -0.39, *p* = 0.0090), SG (rho = -0.48, *p* = 0.000002), and RYGB groups (rho = -0.71, *p* = 0.000033).

There was a positive association between elevated adiponectin and SHBG in the SG (rho = 0.35, *p* = 0.0011), RYGB (rho = 0.81, *p* = 0.000001), and Diet groups. Adiponectin was also positively associated with serum osteocalcin in the Diet (rho = 0.42, *p* = 0.0018), SG (rho = 0.25, *p* = 0.014), and RYGB groups (rho = 0.54, *p* = 0.0030). Positive associations were observed between adiponectin and undercarboxylated osteocalcin in the diet (rho = 0.40, *p* = 0.027), LAGB (rho = -0.45, *p* = 0.047), SG (rho = 0.22, *p* = 0.070), and RYGB groups (rho = 0.67, *p* = 0.012).

##### Undercarboxylated osteocalcin

Fat mass was negatively correlated with ucOC in Diet (rho = -0.68, *p* = 0.00013), SG (rho = -0.46, *p* = 0.00017), and RYGB groups (rho = -0.55, *p* = 0.050). No significant correlations were found between ucOC or osteocalcin and lean mass in any of the surgical groups.

#### Associations between changes in body composition and hormonal indices examined in random intercept mixed effect models over 36 months

##### Adiponectin

Between 0 and 36 months, after adjusting for procedure, age, and sex, a twofold increase in adiponectin was associated with a 10% reduction in total fat mass (95% CI: -18%, -2.5%), a relationship not observed in the unadjusted model (*p* = 0.41). Lean mass declined by 5.0% (95% CI: -9.4%, -2.4%) in the adjusted model and by 13.4% (95% CI: -17.4%, -9.3%) in the unadjusted model.

##### Undercarboxylated osteocalcin

From 0 to 36 months, each twofold increase in ucOC was associated with a 7.7% reduction in total fat mass (95% CI: -12.4%, -3.0%) after adjustment for procedure, age, and sex, and 8.0% (95% CI: -12.8%, -3.2%) in the unadjusted model. Lean mass declined by 3.7% (95% CI: -5.0%, -1.3%) in the adjusted model, with no significant change observed in the unadjusted model (*p* = 0.079).

No significant associations were found between changes in body composition and changes in total osteocalcin or SHBG over time.

## Discussion

Extending our previous analyses of this cohort, the present study examined long-term body composition changes and hormonal predictors of lean mass loss over three years following bariatric procedures [21, 23]. Over 36 months, RYGB resulted in greater total fat mass loss than Diet al.one, likely reflecting reduced visceral fat, as indicated by a significant waist circumference decrease.

Concerningly, all surgical groups experienced a progressive lean mass loss, with RYGB showing sustained decline even during the weight plateau phase, culminating in a 14% total loss. At 36 months, lean mass loss accounted for 21.5% of total weight lost for RYGB, aligning with previous reports (23.4%) and reaching 31% in the SG group [24]. Notably, the proportional lean mass loss increased over time in SG and RYGB groups but remained stable in LAGB and Diet cohorts.

After adjusting for age and sex, all surgical groups demonstrated significantly greater reduction in lean mass than the Diet group, despite similar protein intake within the recommended range (10%–35% of total energy) although transient increases in protein intake during the first three postoperative months were not maintained thereafter) [25].

These findings are consistent with prior reports of 13 ± 6% reduction in lean mass after RYGB, accompanied by a 48% decrease in fat mass and a 30% reduction in total body weight within 12 months with improvements in their muscle strength and physical performance [26].

Most concerningly, we observed that women lost an average 30% more lean mass than men and lean mass loss tended to increase with age. The female-only sensitivity analysis demonstrates even larger lean mass losses—especially after RYGB—confirming and strengthening our primary conclusion that women are disproportionately affected. This is consistent with previous research of women experiencing higher relative reduction in lean mass compared to men (− 15.8 vs.−8.6%)^(27)^, The greater decline in fat-free mass observed in women and older adults aligns with known physiological differences in muscle biology and responses to weight loss. Women typically begin bariatric surgery with lower baseline muscle mass and lower anabolic hormone levels (e.g., testosterone and IGF-1), predisposing them to proportionally greater lean mass loss during rapid postoperative weight reduction [27, 28]. Age-related anabolic resistance reduced mitochondrial efficiency, chronic low-grade inflammation, and decreased physical activity with reduced capacity to maintain muscle further amplify FFM decline in older adults [29]. These findings highlight the potentially harmful impact of bariatric procedures on body composition, particularly in older adults, most notably postmenopausal women, where fat-independent lean mass loss may heighten the risk of frailty [24].

Although no clear threshold exists, fat-free mass loss should ideally remain below 22% of total weight loss, though this may vary by demographic and metabolic factors [30, 31]. FFM changes also depend on diet, physical activity, baseline adiposity, and hormonal responses, underscoring the absence of a universal reference point across obesity interventions loss [32].

Excessive lean mass loss over time may impair muscle function, lower metabolic rate, and increase frailty risk. Alarmingly, a previous study reported that the sarcopenia risk score, increased from 8% to 32% within one year post SG [33]. Given that the most substantial lean mass decline typically occurs within first three months post-surgery, perioperative strategies such as ensuring adequate protein intake (generally above 60–80 g/day) along with resistance training are crucial to preserving muscle mass [24].

Serum adiponectin, a hormone derived from fat tissue, increased significantly during the first 12 months across all surgical groups, with smaller increments between 12 and 36 months. Adiponectin levels were inversely associated with fat mass changes in all surgical groups as well as with lean mass loss in SG and RYGB and Diet groups. These associations between increased adiponectin and ucOC and fat and lean mass loss were further confirmed through multivariate mixed-model analysis, while no such relationship was observed with total osteocalcin or SHBG. The magnitude of this association was modest, indicating that endocrine factors likely play only a secondary role, with mechanical unloading from substantial fat loss and postoperative changes in energy balance remaining the primary drivers of lean mass decline.

A meta-analysis of 557 sarcopenic subjects found that low muscle mass is linked to higher serum adiponectin levels [16]. A recent study in patients with metabolic syndrome and obesity found higher adiponectin levels in those with low skeletal muscle mass, particularly in older individuals [34]. Potential mechanisms included intramuscular adipose tissue fat accumulation affecting adiponectin expression, downregulated adiponectin receptor signalling, and inflammation driven muscle catabolism [35–37]. Given adiponectin’s regenerative and anti-inflammatory roles, its association with lean mass loss ‒ despite the lack of direct muscle measurements in the present study ‒ suggests it may be a potential biomarker of muscle damage in low muscle mass, warranting further investigation.

Contrary to previous reports, we found no consistent relationship between changes in osteocalcin and alterations in body composition [38]. However, a significant association was observed between ucOC levels and fat mass changes across the Diet, SG and RYGB groups and for the study total cohort accounting for age and sex. This finding supports the role of ucOC in regulating fat metabolism ‒directly by influencing adipocyte signalling and indirectly by stimulating insulin secretion from pancreatic β-cells [7] as well as in modulating muscle function and overall body physiology [39, 40].

Congruent with other studies, the observed marked reduction in BMI and fat mass led to a long term, over 36 months, improved lipid profiles for SG and RYGB but not for LAGB or Diet groups at 24 months [41]. These favourable changes in lipids observed in our patients likely reflect surgery-specific hormonal effects on insulin sensitivity in muscle and adipose tissue, in addition to reduced intake and absorption [42]. Notably a previous study of RYGB and SG bariatric patients linked improvements in dyslipidaemia with increased apolipoprotein C and adiponectin secretion [14, 43].

A key strength of this study is its longitudinal design, with repeated assessments of weight and body composition via DXA, alongside measurements of fat-derived (adiponectin) and osteoblast-derived (osteocalcin and undercarboxylated osteocalcin) hormones over three years. By adjusting all models for baseline body size, the estimated group-specific differences in fat and lean mass trajectories over 36 months are interpreted independently of the baseline disparities between bariatric and Diet groups. Bariatric surgery provided a time-controlled model of adipose and lean mass regulation, requiring adaptation to rapid mechanical and endocrine changes. This setting enabled us to examine how body composition changes relate to total weight loss and to circulating markers of energy homeostasis, including the underexplored ucOC, which—along with adiponectin—may serve as a marker of lean mass loss. Comparison of surgical and non-surgical interventions further highlighted differences in body composition and hormonal responses, offering insights into these regulatory pathways in humans.

However, our study is limited by its non-randomized design. For ethical reasons, participants were not randomly assigned to bariatric surgery or dietary intervention; instead, selection was based on patient and physician preference using the DiaRem score. Although the relatively small RYGB sample size limits precision, the large, standardised effect sizes and high post hoc power (> 90% for the observed RYGB–Diet difference in lean mass change) support the robustness of the direction of the findings; these findings still should be considered exploratory. While dietary intake and physical activity were assessed in the broader cohort, they were not included in the present endocrine-focused models therefore residual confounding from lifestyle factors cannot be excluded. Consequently, our results do not establish causality between hormonal changes and changes in body composition due to potential covariate imbalance, which we mitigated by adjusting for baseline variables. A limitation of this study is the absence of DXA data from the early postoperative period (0–3 months) as the study was designed to focus on long-term (6–36 month) trajectories and hormonal predictors of sustained lean mass change. Finally, as appendicular lean mass and functional assessments recommended by the European Working Group on Sarcopenia in Older People 2 (EWGSOP2) were not included in the study protocol our findings cannot be interpreted within a sarcopenia diagnostic framework. However, the magnitude of lean mass loss observed may increase susceptibility to sarcopenia, particularly among older adults and women.

## Conclusion

In summary, this study suggests that while bariatric procedures such as SG and RYGB are more effective than LAGB or dietary interventions in promoting sustained fat mass loss over three years, they may adversely affect body composition due to ongoing lean mass decline. Notably, all surgical groups experienced further lean mass loss after the first postoperative year, with a significant reduction in the RYGB group despite weight stability. This effect was more pronounced in women and became increasingly evident with age although the sex-specific findings should be interpreted with caution due to the smaller number of male participants. Therefore, regular monitoring of body composition, nutritional status, and bone health are essential to ensure long-term positive health outcomes.

The present study demonstrated the crosstalk between skeleton and adipose tissue as a homeostatic feedback system, with adipokines and osteokines actively linking both tissues via as adipose-bone axis.

It has highlighted a potential link between surgically induced hormonal changes including raised adiponectin, and alterations in body composition, possibly via synergy with ucOC however we acknowledge that these relationships are observational and should not be interpreted as causative.

Future randomized studies are needed to evaluate the effects of bariatric surgery-induced weight loss on sarcopenia risk and to determine whether targeted interventions can preserve lean mass while retaining metabolic benefits. Further research is warranted, especially in older and female populations, to clarify the long-term effects on muscle health.

## Supplementary Information

Below is the link to the electronic supplementary material.

Supplementary Material 1

Supplementary Material 2

## Data Availability

All data generated or analysed during this study are included in this published article (and its supplementary information files).
