# Supplementary figures and images for: Long-term body composition changes after bariatric surgery and their association with fat- and bone-derived hormones
Source: Endocrine. 2026 Mar 9;91(1):89. doi: 10.1007/s12020-026-04564-0 (PMC12971941; doi:10.1007/s12020-026-04564-0)

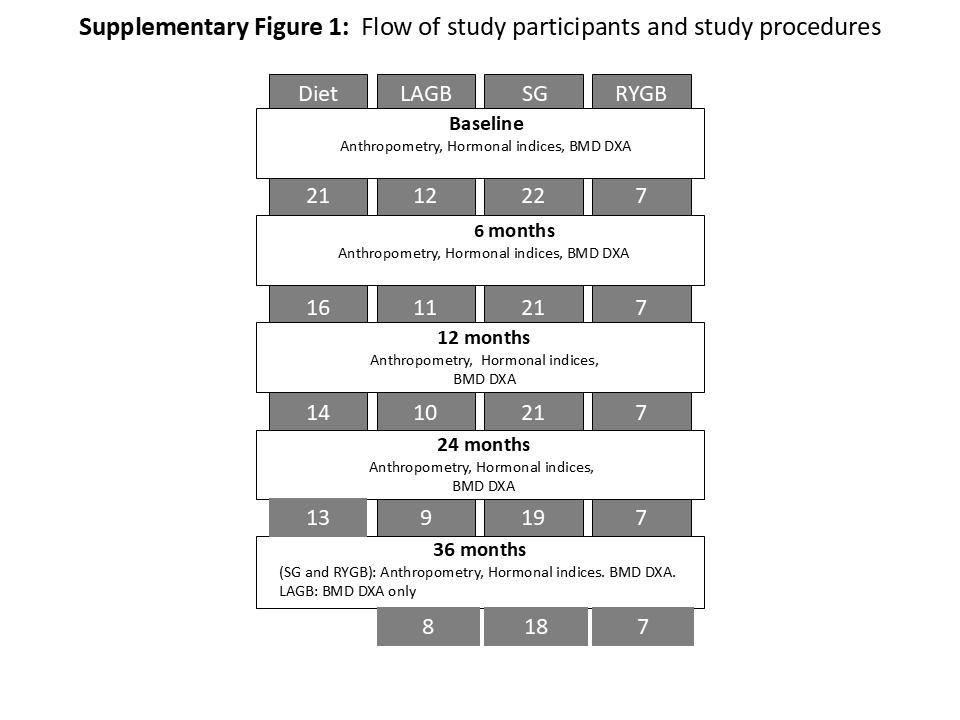

Supplement: Supplementary file 1 — Supplementary Material 1 [file 12020_2026_4564_MOESM1_ESM.tif]
