# Supplementary material for: Long-term body composition changes after bariatric surgery and their association with fat- and bone-derived hormones
Source: Endocrine. 2026 Mar 9;91(1):89. doi: 10.1007/s12020-026-04564-0 (PMC12971941; doi:10.1007/s12020-026-04564-0)
Supplement: Supplementary file 2 — Supplementary Material 2 [file 12020_2026_4564_MOESM2_ESM.docx]

Supplementary Table 1: Lipid Profile Data by Study Timepoint

| **Variables** | **Baseline** | **12 months** | **36 months** |
| --- | --- | --- | --- |
| Diet |  |  |  |
| Total cholesterol (mmol/L) | 5.5 (4.5‒6.25) | 5.3 (4.5‒5.9) |  |
| HDL cholesterol (mmol/L) | 1.3 (1.25‒1.45) | 1.2 (1.2‒1.4) |  |
| Triglycerides (mmol/L) | 1.45 (1.35‒2.3) | 1.3 (1.0‒1.7) |  |
| LDL cholesterol (mmol/L) | 3.1 (2.85‒4.0) | 3.5 (2.7‒3.9) |  |
| LAGB |  |  |  |
| Total cholesterol (mmol/L) | 4.5 (4.0‒5.3) | **4.35 (3.6‒5.45)** |  |
| HDL cholesterol (mmol/L) | 1.2 (0.9‒1.3) | **1.25 (1.1‒1.45)** |  |
| Triglycerides (mmol/L) | 1.0 (0.7‒1.5) | 1.0 (0.55‒1.1) |  |
| LDL cholesterol (mmol/L) | 3.2 (2.5‒3.6) | 2.85(2.2‒3.45) |  |
| SG |  |  |  |
| Total cholesterol (mmol/L) | 4.5 (4.2‒5.3) | 4.4 (4.2‒5.3) | 4.35 (4.3‒5.5) |
| HDL cholesterol (mmol/L) | 1.2 (1.0‒1.3) | **1.5 (1.2‒1.8)** | **1.5 (1.4‒2.2)** |
| Triglycerides (mmol/L) | 1.6 (1.0‒1.9) | **1.1 (0.6‒1.3)** | **0.85 (0.7, 1.3)** |
| LDL cholesterol (mmol/L) | 2.9 (2.1‒3.6) | 2.6 (2.1‒3.1) | **2.35 (1.8‒3.5)** |
| RYGB |  |  |  |
| Total cholesterol (mmol/L) | 4.8 (4.6‒4.8) | 3.8 (3.7‒4.2) | 3.8 (3.7‒4.2) |
| HDL cholesterol (mmol/L) | 1.2 (0.7‒1.4) | 1.3 (0.8‒1.5) | 1.3 (0.8‒1.5) |
| Triglycerides (mmol/L) | 1.3 (0.7‒5.1) | 1.0 (0.4‒1.6) | 1.0 (0.4‒1.6) |
| LDL cholesterol (mmol/L) | 3.05 (3‒3.1) | 2.2 (2.1‒2.5) | 2.2 (2.1‒2.5) |

Results presented as median values with interquartile range. Bold type denotes statistically significant changes relative to baseline. **Abbreviations**: LAGB, laparoscopic gastric banding; RYGB, Roux-en-Y gastric bypass: SG, sleeve gastrectomy
